# *Aloysia citrodora* Polyphenolic Extract: From Anti-Glycative Activity to In Vitro Bioaccessibility and In Silico Studies

**DOI:** 10.3390/nu18010115

**Published:** 2025-12-29

**Authors:** Giulia Moretto, Raffaella Colombo, Stefano Negri, Stefano Alcaro, Francesca Alessandra Ambrosio, Giosuè Costa, Adele Papetti

**Affiliations:** 1 Drug Sciences Department, University of Pavia, 27100 Pavia, Italy; giulia.moretto01@universitadipavia.it (G.M.); raffaella.colombo@unipv.it (R.C.); 2 National Biodiversity Future Center (NBFC), 90133 Palermo, Italy; stefano.negri@univr.it; 3 Department of Biotechnology, University of Verona, 37134 Verona, Italy; 4 Dipartimento di Scienze della Salute, Università degli Studi “Magna Græcia” di Catanzaro, Campus “S. Venuta”, Viale Europa, 88100 Catanzaro, Italy; alcaro@unicz.it (S.A.); ambrosio@unicz.it (F.A.A.); gcosta@unicz.it (G.C.); 5 Net4Science Academic Spin-Off, Università “Magna Græcia” di Catanzaro, Campus “S. Venuta”, 88100 Catanzaro, Italy; 6 Associazione CRISEA—Centro di Ricerca e Servizi Avanzati per l’Innovazione Rurale, Località Condoleo, 88055 Belcastro, CZ, Italy

**Keywords:** *Aloysia citrodora* edible leaf extract, natural anti-glycative agent, RAGE, aging-related diseases, bioaccessibility, molecular modeling, verbascoside

## Abstract

**Background:** The in vivo accumulation of Advanced Glycation End products (AGEs) is associated with the development of several chronic aging-related and degenerative diseases, as they alter protein structures and activate oxidative and inflammatory processes through interactions with the receptor for AGEs (RAGE). Plant secondary metabolites play a key role in counteracting the glycation process through various mechanisms of action. Therefore, *Aloysia citrodora* leaf polyphenolic extract could represent a source of anti-glycative compounds. **Methods:** The methanolic extract was characterized by RP-HPLC-DAD-MS^n^, and its anti-glycative properties were investigated using several in vitro assays mimicking the different steps of the glycation reaction. In parallel, molecular docking studies were carried out to evaluate potential interactions between the identified metabolites and RAGE. Furthermore, *A. citrodora* metabolites’ stability under simulated in vitro digestion was assessed, and the anti-glycative activity of the bioaccessible fraction was investigated. **Results:** *A. citrodora* extract, rich in iridoid glycosides, phenylethanoid glycosides, and flavones, strongly inhibited AGE formation (from 10% to 100%) in both the middle and end step of the reaction and had high methylglyoxal and glyoxal trapping capacity. However, the digestion process affected extract stability, particularly under intestinal conditions, yielding an overall bioaccessibility of about 40% and leading to a subsequent reduction in anti-glycative properties. Finally, molecular modeling analysis highlighted the ability of the studied metabolites to bind RAGE. **Conclusions:** *A. citrodora* represents a promising source of natural anti-glycative agents with potential applications as food ingredients. However, it is essential to improve the extract bioaccessibility and to preserve its anti-glycative properties by developing a suitable formulation.

## 1. Introduction

*Aloysia citrodora* Paláu, commonly known as lemon verbena, is an aromatic perennial plant belonging to the Verbenaceae family that is native to South America, particularly Argentina and Chile. The plant is well known for the essential oil obtained from its leaves, which is rich in monoterpenes and sesquiterpenes and which gives it its characteristic lemon aroma; this has favored its spread throughout southern Europe (including Italy), Africa, and the Mediterranean region [1]. Traditionally, lemon verbena leaf infusion is used to treat gastrointestinal disorders, fever, asthmatic spasms, flatulence, cramps, and diarrhea, as well as a remedy for nervous system conditions such as stress, anxiety, and insomnia. Today, it is also consumed as a refreshing and tonic beverage [1,2]. The essential oil is widely used as a flavoring and perfuming agent in the food, beverage, cosmetic, and perfume industries. In addition, it is widely used as a drug in traditional medicine due to its well-known diuretic, antispasmodic, sedative, and antimicrobial properties [1,2]. Apart from the volatile fraction, *A. citrodora* contains a wide range of bioactive secondary metabolites, including iridoid glycosides, phenylethanoid glycosides, phenylpropanoid glycosides, and flavonoid derivatives [3]. These compounds have been associated with numerous biological activities, including antioxidative, anti-inflammatory, antimicrobial, hepatoprotective, neuroprotective, and anxiolytic properties, and their potential role in the treatment of metabolic and cardiovascular disorders has been highlighted [2]. Therefore, the combination of ethnobotanical and scientific knowledge makes lemon verbena leaf extract a highly interesting edible ingredient with a broad healthy profile.

Over the past few decades, interest in plant-derived bioactive compounds has grown considerably, as well as their potential application as active ingredients in food supplements aimed at promoting health and preventing the risk of developing chronic diseases. Several studies support the beneficial role of secondary metabolites in diabetes mellitus, cardiovascular disorders, neurodegeneration, and chronic inflammation [4]. One of the main risk factors in the development of chronic degenerative disorders is the formation and accumulation of Advanced Glycation End products (AGEs), which are generated by non-enzymatic reactions between reducing sugars and proteins under conditions of hyperglycemia and oxidative stress [5]. AGEs can alter protein structure and function through crosslinking or by interacting with their cellular receptor (Receptor for Advanced Glycation End products; RAGE), thus activating inflammatory and oxidative pathways [6]. Secondary metabolites have been reported to counteract AGEs formation through multiple mechanisms, including metal ion chelation, a reduction in free radical formation, the trapping of reactive dicarbonyl compounds, and the protection of amino groups from glycation. In addition, some compounds can form RAGE–ligand complexes, thereby modulating receptor activity [7,8]. In this context, natural compounds with anti-glycative properties represent a valid alternative to synthetic inhibitors, which are often associated with adverse effects. Thus, the search for new edible plant extracts that are rich in secondary metabolites could open promising perspectives for the identification of new natural anti-glycative agents as well as of new food ingredients [9,10]. However, the effectiveness of secondary metabolites largely depends on their stability during digestion, since they can undergo chemical or structural changes in the gastrointestinal tract, which can significantly affect their bioactivity [11]. For this reason, assessing bioaccessibility, defined as the fraction of a compound released from the food matrix and available for absorption, is essential in evaluating the efficacy of bioactive compounds [12].

The present study investigated the capacity of *A. citrodora* edible leaf extract to inhibit AGEs formation. Several in vitro assays were performed to evaluate its anti-glycative activity at different steps of the glycation process [13,14,15]. In parallel, the phytochemical profile of the extract was characterized using RP-HPLC-DAD-ESI-MS^n^, and molecular modeling analyses were conducted to elucidate the binding mode and the interactions established between the identified compounds and RAGE. Additionally, the stability of *A. citrodora* metabolites under simulated in vitro digestion was assessed, and the anti-glycative activity of the bioaccessible fraction was investigated [16,17].

Overall, this integrated approach provided valuable insights for identifying novel natural anti-glycative agents and for supporting the design of new food ingredients for the prevention or management of chronic aging-related and degenerative diseases. In particular, the research aimed to address a knowledge gap concerning natural compounds that are able to act on multiple aspects of the glycation reaction, both by reducing AGEs formation and by targeting RAGE, as no data are currently available about the anti-glycative properties of *A. citrodora* or about the effect of in vitro digestion on its leaf extract. Therefore, these bioaccessibility studies contribute to understanding how gastrointestinal digestion may affect the stability and the biological properties of *A. citrodora*, thereby supporting the definition of effective formulation strategies aimed at ensuring stability, absorption, and biological efficacy of plant-derived ingredients.

## 2. Materials and Methods

### 2.1. Plant Material

*Aloysia citrodora* Paláu (Verbenaceae family) leaves were collected in triplicate (50 leaves per replicate) from the aerial parts of a single *Aloysia citrodora* plant grown at the Botanical Garden of Padua (Italy) during its flowering phase. Immediately after collection, samples were flash-frozen in liquid nitrogen and ground to a fine powder using an A11 basic analytical mill (IKA-Werke, Staufen, Germany). Extraction was performed by adding LC-MS grade methanol (Honeywell, Seelze, Germany) to the frozen powder at a ratio of 1:10 (*w*/*v*). The mixture was briefly vortexed (30 s) and sonicated in an ice-cooled ultrasonic bath (SOLTEC, Milan, Italy) at 40 kHz for 10 min. Then, the suspension was centrifuged at 14,000× *g* for 10 min at 4 °C. The resulting supernatants were divided into 1 mL aliquots (each equivalent to the extract derived from 100 mg of fresh plant material), dried using a Speed-Vac system (He-to-Holten, Frederiksborg, Denmark), and stored at −20 °C until further use. Each aliquot was reconstituted and diluted as required by the specific experimental procedures before being used.

### 2.2. Reagents

HPLC grade acetic acid, formic acid, acetonitrile, methylglyoxal (MGO, 40% aqueous solution), glyoxal (GO, 40% aqueous solution), aminoguanidine hydrochloride (AG; purity grade ≥ 98%), nitrotetrazolium blue chloride (NBT; purity grade ≥ 90%), bovine serum albumin (BSA; purity grade ≥ 98%), D-(+)-glucose (GLU; purity grade ≥ 99.5%), D-(−)-fructose (FRU; purity grade ≥ 99%), 5-methylquinoxaline (5-MQ; purity grade ≥ 98%), *o*-phenylenediamine (OPD; purity grade ≥ 98%), sodium dihydrogen phosphate monohydrate (purity grade ≥ 98%), disodium hydrogen phosphate dodecahydrate (purity grade ≥ 99%), sodium azide (purity grade ≥ 99.5%), Type VI porcine pancreatic α-amylase, pepsin from porcine gastric mucosa (≥400 U/mg), bile extract porcine, and pancreatin (8× USP) from porcine pancreas were provided by Merck Life Science S.r.l. (Milan, Italy). Ethanol (96%) was supplied by Carlo Erba (Milan, Italy). LC-MS grade methanol was provided by Honeywell (Seelze, Germany). Water was obtained from a Millipore Direct-QTM system (Merck Millipore, Milan, Italy). Verbascoside was purchased from Extrasynthese (purity grade ≥ 98%; Genay, Rhone, France).

### 2.3. Evaluation of Fructosamine Formation

The formation of Amadori products was evaluated through the quantification of fructosamine content using the nitroblue tetrazolium (NBT) colorimetric assay, following the protocol described by Maietta et al. [13,18]. Each aliquot of dried extract was dissolved in 2 mL of an EtOH:H_2_O mixture (1:3, *v*/*v*) and diluted with phosphate buffer (100 mM, pH 7.4, containing 0.02% NaN_3_), yielding final concentrations of 0.5 and 1 mg/mL, respectively, in the reaction mixtures. The ability of the extract to inhibit fructosamine formation was identified from the inhibition percentage (I%) following this equation:

I% = [1 − (Abs_glycated system+extract_ − Abs_background+extract_)/(Abs_glycated system_ − Abs_background_)] × 100 (1)

where Abs is the absorbance registered at 530 nm (Spectrophotometer Lambda 25, Perkin Elmer, Milan, Italy) and the background is related to a BSA solution.

### 2.4. Evaluation of MGO and GO Trapped by the RP-UHPLC-DAD Method

The undigested and digested extract’s trapping capacity toward GO and MGO was determined following the method described by Mesías et al. [14].

Each aliquot of dried extract was dissolved in 2 and 1 mL of an EtOH:H_2_O mixture (1:3, *v*/*v*) and properly diluted with phosphate buffer (100 mM, pH 7.4, 0.02% sodium azide) to obtain the final concentrations of 0.5 and 1 mg/mL, respectively. 5-MQ (6.5 mM) was also added as an internal standard.

The digested extract was dissolved in 4 mL of H_2_O and then diluted 1:10, as previously described, resulting in a final concentration of 0.125 mg/mL.

Then, the reaction mixture was incubated at 37 °C for 24, 48, 72, and 96 h in the presence or absence of the undigested or digested extract. Quantification of residual GO and MGO as quinoxaline derivatives was performed by RP-UHPLC-DAD according to the method described by Moretto et al. GO and MGO trapped by the extracts was assessed as described by Moretto et al. [19].

### 2.5. Evaluation of Vesperlysine-like and Argpyrimidine-like AGE Formation

An in vitro model system consisting of a protein (BSA) and a glycative agent (MGO or FRU) was used to assess the anti-glycative activity in the undigested extract at both the middle and final steps of the glycation reaction, as well as in the digested extract in the presence of FRU [13,14,15,18]. For the middle step, BSA was incubated with MGO at 37 °C for 1, 4, and 7 days in the absence or presence of the undigested extract (0.5 and 1 mg/mL in the reaction mixture) to evaluate the formation of Argpyrimidine-like and Vesperlysine-like AGEs. In contrast, the extract’s ability to inhibit Vesperlysine-like AGEs formation in the final step was evaluated by incubating BSA with FRU for 4, 7, and 14 days with or without the undigested and digested extract (1 mg/mL and 0.125 mg/mL, respectively). Extract solutions were prepared as described in Section 2.3. On the other hand, the digested extract was dissolved in 4 mL of H_2_O and diluted 1:2, resulting in a final concentration of 0.125 mg/mL in the mixture reaction. AG (0.5 mg/mL) was used as a positive control. AGE formation was monitored by measuring the fluorescence intensity (FI) at excitation/emission wavelengths of 335/440 nm for Argpyrimidine-like AGEs and at 370/440 nm for Vesperlysine-like AGEs (Spectrofluorometer L550B; Perkin Elmer, Milan, Italy). The inhibitory effect (inhibition percentage; I%) of the extract on AGE formation was calculated as reported by Maietta et al. [18], using the following equation:

I% = [1 − ((FI_glycated system (BSA+FRU or MGO+extract)_ − FI_background (BSA+extract)_)/(FI_glycated system (BSA+FRU or MGO)_ − FI_background (BSA)_))] × 100. (2)

### 2.6. Analysis of the Metabolic Profile by RP-HPLC-DAD-ESI-MS^n^ Method

The identification and quantification of compounds in the *A. citrodora* leaf methanolic extract (1 mg/mL) were performed by RP-HPLC-DAD-ESI-MS^n^. The analyses were conducted using a Thermo Finnigan Surveyor Plus HPLC system (Thermo Fisher Scientific, Waltham, MA, USA), equipped with a quaternary pump, a UV–Vis photodiode-array detector, an autosampler, and a vacuum degasser, coupled to an LCQ Ad-vantage Max ion-trap mass spectrometer via an electrospray ionization (ESI) source. Chromatographic separation was performed on a Gemini^®^ C18 analytical column (150 × 2.0 mm i.d., 5 μm particle size; Phenomenex, Torrance, CA, USA) operating at a constant flow rate of 0.3 mL/min with a 20 μL injection volume. The mobile phase consisted of 0.1% aqueous formic acid (solvent A) and 0.1% formic acid in acetonitrile (solvent B) with the following gradient program: 0–3 min, 2% B; 3–5 min, 2–13% B; 5–9 min, 13% B; 9–12 min, 13–18% B; 12–15 min, 18–30% B; 15–25 min, 30–45% B; 25–32 min, 45–60% B; 32–37.5 min, 60–80% B; 37.5–40 min, 80–98% B; 40–45 min, 98–2% B; 45–55 min, 2%. The ion trap operated under a data-dependent acquisition, full scan (50–2000 *m*/*z*), and MS^n^ mode to obtain fragment ions, using a normalized collision energy of 30% and an isolation width of 3 *m*/*z*. Verbascoside (10 ppm in 0.1% formic acid aqueous solution—0.1% formic acid in acetonitrile solution; 50:50, *v*/*v*) was used to optimize the mass spectrometric parameters by flow injection analysis. Specifically, ESI source conditions in the negative ionization mode were as follows: sheath gas flow rate, 20 arbitrary units (AU); auxiliary gas flow rate, 10 AU; spray voltage, 4 kV; capillary temperature, 300 °C. Acquisition and processing were performed using Thermo Fisher Scientific Xcalibur 2.1 software. The identification of secondary metabolites was based on the comparison of retention times, the UV-Vis spectra, and MS fragmentation patterns with those of authentic standards, when available, or with literature data. Verbascoside quantification was performed using the calibration curve of the corresponding standard (and quantified as µg/mL), and the verbascoside-related derivatives were quantified as verbascoside equivalents (VE µg/mL). The LC-MS method was validated in accordance with the International Conference on Harmonization (ICH) guidelines Q2(R2) (2022), considering linearity, accuracy, precision, the limit of detection (LOD), and the limit of quantification (LOQ) [20]. Linearity was evaluated according to the external standard method, using a calibration curve prepared with verbascoside at five concentration levels (0.5–10 µg/mL), each analyzed in triplicate. The peak area (y) of the [M-H]^−^ ion was plotted against the corresponding theoretical concentrations to construct the calibration curve, and linear regression analysis was performed using the least-squares method. A correlation coefficient (R^2^) of at least 0.9900 was established as the acceptance criterion. LOD and LOQ were defined based on signal-to-noise (S/N) ratios, corresponding to approximate S/N values of 3 for LOD and 10 for LOQ. Precision was determined by analyzing standard solutions in triplicate at each concentration level, evaluating both the intra-day (same day) and inter-day (across three separate days) repeatability. Precision results were expressed as the relative standard deviation (RSD%) of the replicate measurements. Accuracy was assessed through recovery experiments at three concentration levels (0.5, 1, and 10 µg/mL), corresponding to low, medium, and high concentrations. For each level, samples were analyzed in triplicate, and recovery was calculated as the percentage ratio between the measured and theoretical concentrations of the compound. Verbascoside stock solution (1 mg/mL) was prepared by dissolving 1 mg of standard in 1 mL of methanol. Samples used for the RP-HPLC-DAD-ESI-MS^n^ analyses were prepared by dilution of the stock solution with 0.1% formic acid aqueous solution and 0.1% formic acid in acetonitrile (50:50 *v*/*v*).

### 2.7. In Vitro Simulated Gastrointestinal Digestion

Static in vitro gastrointestinal digestion was performed according to the standardized INFOGEST protocol [16,17]. Simulated digestive fluids, including simulated salivary fluid (SSF), simulated gastric fluid (SGF), and simulated intestinal fluid (SIF), were prepared following the same protocol. *A. citrodora* extract (1 mg/mL) digestion was conducted in triplicate. In parallel, a blank sample (consisting of simulated digestive fluids and enzymes with water in place of extract) and a control sample (consisting of plant extract with digestive fluids but without enzymes or bile salts) were carried out under the same conditions. The supernatants obtained from each digestion step for the extract (oral digested, OD; gastric digested, GD; intestinal digested, ID) and for the blank and control sample (oral control, OC; gastric control GC; intestinal control, IC) were subjected to bioaccessibility studies. Additionally, the digested extract, along with the control and blank samples, was evaluated for its ability to trap MGO and GO and to inhibit AGEs formation in the BSA-FRU model system, as detailed in Section 2.4 and Section 2.5.

### 2.8. Evaluation of Extract Bioaccessibility

The effect of digestion on the *A. citrodora* extract’s secondary metabolites was evaluated by assessing their bioaccessibility. Samples obtained at each digestive stage (oral, gastric, and intestinal) were freeze-dried and analyzed using RP-HPLC-DAD-ESI-MS^n^, as described in Section 2.6, to evaluate and compare the secondary metabolites’ qualitative and quantitative profile before and after digestion. All oral, gastric, and intestinal samples (digested, blank, and control) collected after in vitro digestion were initially dissolved in water (1, 2, and 4 mL of H_2_O, respectively) and subsequently diluted 1:10 with the mobile phase to reach final concentrations of 0.5, 0.25, or 0.125 mg/mL, according to the respective dilution factors for each digestion step. Before the chromatographic analysis, samples were filtered through 0.45 µm regenerated cellulose (RC) membranes (Phenomenex^®^, Torrance, CA, USA) to remove particulate matter. Blank solutions were also analyzed to subtract the potential background signals originating from enzymes, buffers, or other reagents involved in the digestion process. Bioaccessibility was expressed as a bioaccessibility index percentage and calculated as reported by Colasanto et al. [21]:

(3) $$Bioaccessibility\;index\left(\%\right)=\;\frac{\;digested\;sample\;}{undigested\;sample}\;\times 100$$

As indicated above, the bioaccessibility index (%) was calculated as the ratio between the metabolite content (µg/mL or VE µg/mL) in the sample after each digestive phase and that in the extract before digestion.

### 2.9. Molecular Modeling Studies

The crystal structure of human RAGE (PDB code 4P2Y) retrieved from the Protein Data Bank (PDB) [22] was used for the molecular modeling studies. The receptor structure was properly refined through the Protein Preparation Wizard tool (Schrödinger Release 2020-4), using OPLS_2005 as the force field [23]. In detail, residual crystallographic buffer components were removed, missing side chains were built using the Prime module (Schrödinger Release 2020-4), [24] hydrogen atoms were added, and side-chain protonation states at pH 7.4 were assigned.

To explore the ensemble of RAGE conformations, molecular dynamics (MD) simulation was conducted using Desmond package v. 4.2 [25]. The system was immersed in an orthorhombic box of TIP4P water molecules extending at least 10 Å from the protein, and counter ions were added to neutralize the system charge. The temperature was set at 300 K, and the NPT ensemble was selected. The MD simulation was carried out for 200 ns.

After the molecular dynamics simulations, the obtained trajectory was clustered based on the structural similarity criteria. The clustering of the trajectory was performed with respect to the root mean square deviation (RMSD), resulting in four distinct representative clusters. The representative structure from the most populated cluster was chosen for further docking analyses, as it corresponds to the most frequently sampled and, thus, lowest free-energy conformational state under the simulation conditions. Therefore, it can be considered the most stable and probable conformation of the protein and the most suitable for evaluating the binding mode of the studied compounds.

The library of metabolites was prepared using the LigPrep tool (Schrödinger Release 2020-4) [26]. In detail, hydrogens were added, salts were removed, and ionization states were calculated using Epik (Schrödinger Release 2020-4) at a pH of 7.4 ± 0.2 [27].

Molecular recognition studies were carried out using Glide program v. 8.9 using the standard precision mode and generating 10 poses for the ligand [28]. Finally, MM-GBSA thermodynamic analysis was performed on the best docking pose for each compound to determine the binding free energy (ΔG). The MM-GBSA calculations were run using OPLS_2005 as the force field [29,30,31].

## 3. Results and Discussions

In the context of the National Biodiversity Future Centre (NBFC), funded by the Italian Ministry of University and Research (MUR) through European Union—NextGeneration EU funding, several plant species were sampled to assess their biological properties. A preliminary screening of anti-glycative properties among the available edible plant species was performed, leading to the selection of *A. citrodora* Paláu (Verbenaceae family) as particularly promising for further investigation [19]. It is an edible plant whose leaves as well as flowers and essential oil are included in the Belfrit List, which is a list of plants and their parts that can be used in food preparation in Belgium, France, and Italy.

The metabolic profile of *A. citrodora* leaf extract was firstly chemically characterized, and then the extract’s effects at different steps of the glycation reaction were evaluated before and after simulated in vitro digestion. Simulated digestion also led to bioaccessibility information about the extract components, which were also investigated for their binding mode with RAGE and their theoretical binding free energies by molecular modeling analyses.

### 3.1. Chacterization of the Metabolic Profile of A. citrodora Leaf Extract by RP-HPLC-DAD-ESI-MS^n^ and Method Validation

**Figure 1:**
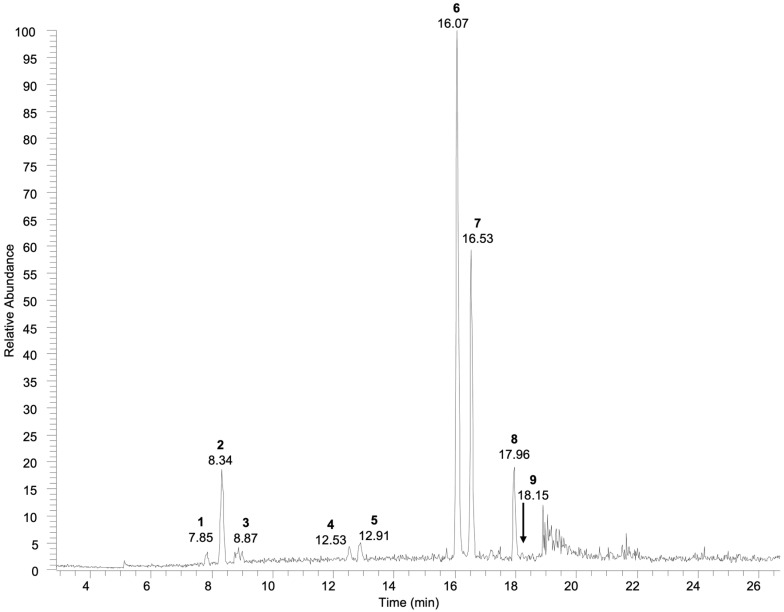
Base-peak chromatogram registered in the negative ionization mode of *A. citrodora* extract (1 mg/mL).

The metabolic profile analysis of the *A. citrodora* leaf extract was performed by RP-HPLC-DAD-ESI-MS^n^ using a data-dependent acquisition mode. Figure 1 presents the base-peak chromatogram recorded in the negative ionization mode. The phytochemical characterization was based on the retention time (Rt), UV-Vis absorption maxima, and mass spectral data obtained in the selected ionization mode, further supported by MS^2^ and MS^3^ fragmentation patterns, and compared with data obtained by analyzing pure reference standards, when available, or with data reported in the literature. Overall, nine metabolites were identified (Table 1), belonging to the following classes: iridoid glycosides, phenylethanoid glycosides, and flavonoids.

Compound 1 showed a molecular ion at *m*/*z* 437, which fragmented to produce the deprotonated ion [M-H]^−^ at *m*/*z* 391, indicating the loss of formate; therefore, further MS^3^ analysis was required for structural elucidation. The fragmentation pattern generated several diagnostic ions, including the fragment at *m*/*z* 229, corresponding to an aglycone after the loss of the sugar moiety, followed by fragments at *m*/*z* 211 [aglycone-H-H_2_O]^−^, *m*/*z* 185 [aglycone-H-CO_2_]^−^, and *m*/*z* 167 [aglycone-H-H_2_O-CO_2_]^−^, which are characteristic of iridoid glycosides. Based on these data, the compound was putatively identified as shanzhiside [32,33]. Similarly, compounds 2 and 3 were identified as geniposidic and loganic acid; these presented a molecular ion at *m*/*z* 419 and 421, respectively, which are adducts with formic acid [M+HCOO]^−^. The fragmentation of these ions generated the deprotonated ion [M-H]^−^ at *m*/*z* 373 and 375, respectively. MS^3^ spectra provided additional diagnostic ions: *m*/*z* 211, 167, 149, and 123 for geniposidic acid, and *m*/*z* 213, 169, 151, and 125 for loganic acid. These fragments corresponded to [aglycone-H-H_2_O]^−^, [aglycone-H-CO_2_]^−^, [aglycone-H-H_2_O-CO_2_]^−^, and [aglycone-H-2CO_2_]^−^, resulting from losses of the glucose moiety and water and from retro Diels–Alder cleavage of the iridoid skeleton. The hemiacetal group of iridoids can readily isomerize, yielding two aldehyde groups, which facilitate bond cleavage between C-4 and C-5 [34,35,36]. In the MS^2^ spectrum of compound 4, a base peak at *m*/*z* 387 and a fragment ion at *m*/*z* 225, attributed to the aglycone moiety, were observed. These data allowed for the identification of rehmaionoside C, which was present in the MS spectrum as a formate adduct [37,38,39]. The molecular ion at *m*/*z* 387 [M-H]^−^, corresponding to compound 5 eluting at 12.91 min, was assigned to tuberonic acid glucoside. Its MS^2^ ions at *m*/*z* 207 and 163 are consistent with the dehydration and decarboxylation, respectively, of the aglycone moiety following the loss of hexoside [40,41]. In the extract, two phenylethanoid derivatives were also detected at 16.07 min and 16.53 min, corresponding to verbascoside (compound 6) and isoverbascoside (compound 7), respectively. Both isobaric compounds had a molecular ion [M-H]^−^ at *m*/*z* 623. Their MS^2^ fragmentation patterns showed the characteristic ion at *m*/*z* 461, resulting from the loss of the caffeoyl moiety [M-H-162]^−^. The identity of verbascoside was confirmed by analysis of the commercial standard compound under the same experimental conditions. Compound 7 was attributed to the isomeric form of verbascoside based on the different selectivity of the two compounds, as previously reported in the literature [42].

Finally, two *O*-methylated flavonoids were identified. Compound 8 at Rt 24.99 min, with a molecular ion [M-H]^−^ at *m*/*z* 299 that fragmented to yield an ion at *m*/*z* 284 resulting from the loss of a methyl group, was putatively attributed to rhamnocitrin [43]. At Rt 25.36 min, a [M-H]^−^ molecular ion at *m*/*z* 329 was detected, showing the characteristic fragment ions at *m*/*z* 314 and 299 deriving from the sequential loss of two methyl groups. It was putatively assigned the jaceosidin structure (compound 9) [44,45]. Overall, the identified metabolites are consistent with those previously reported for *A. citrodora*, supporting the reliability of these findings. As described in the literature, iridoid glycosides, phenylethanoid glycosides, and flavonoids represent the main phytochemical classes. In fact, the metabolites identified have already been described in this plant species, with verbascoside and its isomer recognized as its characteristic compounds [3,46].

The RP-HPLC-DAD-ESI-MS^n^ method developed for the identification of secondary metabolites in *A. citrodora* extract was successfully validated according to ICH guidelines [20] using the external standard method and using verbascoside as the reference compound. Linearity, intra- and inter-day precision and accuracy, as well as LOD and LOQ, were evaluated. Linearity in the concentration range of 0.5–10 µg/mL was demonstrated, with a correlation coefficient (R^2^) of 0.9986. LOD and LOQ values were 0.03 µg/mL and 0.1 µg/mL, respectively, demonstrating high sensitivity. Accuracy results yielded mean recovery values of between 93% and 108% when tested at low, medium, and high concentration levels, indicating an accurate method. Precision, expressed as the relative standard deviation (RSD%), was within the acceptable range, with intra- and inter-day values ranging from 0.4% to 8%. These results supported the reproducibility and overall suitability of the method for both qualitative and quantitative analyses of verbascoside and its isomer identified in *A. citrodora* leaf extract. It was subsequently used to quantify the metabolites both before and after simulated gastrointestinal digestion of the extract.

### 3.2. Evaluation of Inhibitory Effects of A. citrodora Extract on the Glycation Reaction

**Figure 2:**
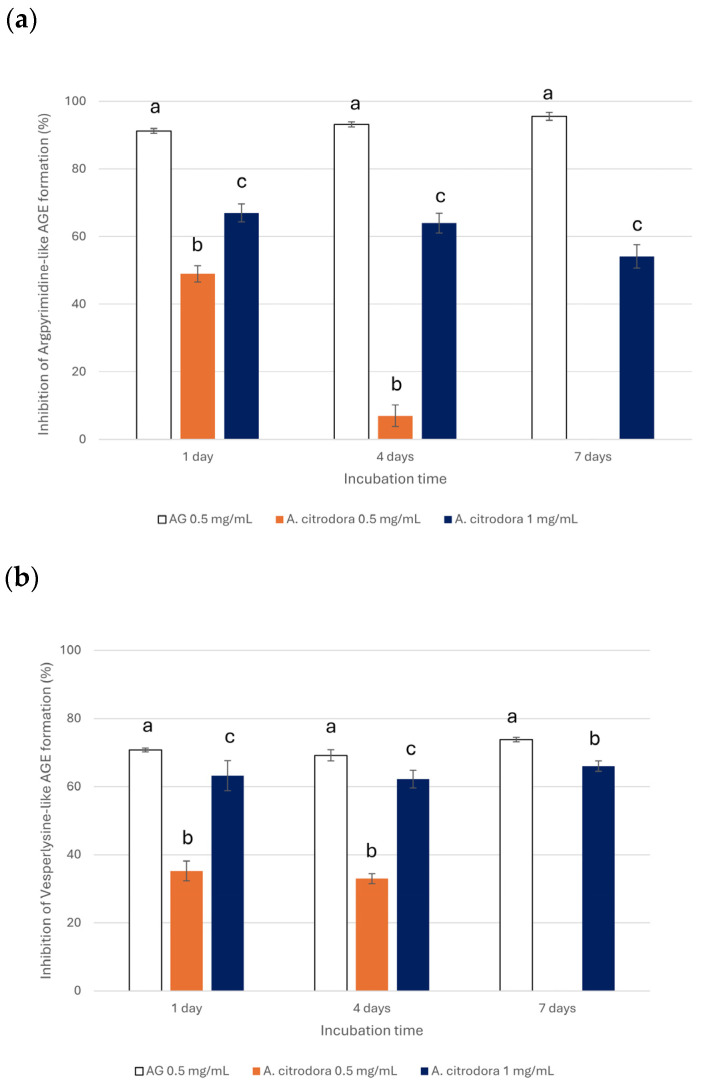
Inhibitory effect of *A. citrodora* extract tested at 0.5 and 1 mg/mL as well as of AG (0.5 mg/mL) against Argpyrimidine-like AGE (**a**) and Vesperlysine-like AGE (**b**) formation in the BSA-MGO system. Statistically significant differences (*p* < 0.05) between the extract and AG are represented by different superscript letters at each incubation time.

AGEs are generated in the glycation reaction and are involved in the development and progression of various chronic aging-related and degenerative diseases, including diabetes type II and neurodegenerative disorders [47]. Over the last few decades, the incidence of such pathologies has dramatically increased, and, therefore, there is increasing interest in the identification of natural compounds that are able to effectively inhibit their formation [4]. In particular, the glycation reaction is a complex, nonenzymatic, multistep process that begins with the reaction between the carbonyl group of reducing sugars, such as GLU or FRU, and the amine group of a protein, forming an unstable Schiff base that then rearranges into more stable products, such as Amadori products. These intermediates may further chemically transform to produce GO and MGO, key components of the middle step of the glycation reaction, which can subsequently interact with amine groups, leading to the formation of AGEs [6]. Therefore, the anti-glycative properties of *A. citrodora* extract were tested at 0.5 mg/mL and 1 mg/mL (concentrations generally used to evaluate the biological properties of plant extracts) and were evaluated at the initial, middle, and final steps of the glycation reaction. The ability of the extract to inhibit Amadori product formation in the initial step of the reaction was assessed using a model system consisting of BSA and GLU incubated at 37 °C over a long period (14 days) to allow for the slow formation of Amadori products resulting from Schiff base rearrangement [48]. Under these conditions, the extract did not exhibit any inhibitory activity at the tested concentrations. In contrast, a BSA-MGO model system was used to evaluate the effect of the extract on the Argpyrimidine-like AGEs (formed by the interaction of MGO with the guanidine group of arginine residues) and Vesperlysine-like AGEs (resulting from glycoxidation) formation in the middle step. The glycative model system was incubated at 37 °C, and the formation of AGEs was monitored after 1, 4, 7 days based on MGO reaction kinetics [18]. AG, a well-known anti-glycative agent, was used as a positive control, and it reduced the formation of Argpyrimidine-like and Vesperlysine-like AGEs by approximately 90% and 70%, respectively, over time. *A. citrodora* extract had a significant inhibitory effect on the formation of Argpyrimidine-like AGEs at both tested concentrations, showing a clear dose- and time-dependent response (Figure 2a). At 0.5 mg/mL, the extract had the highest inhibitory activity (49.11 ± 2.41%) after 1 day, and then the activity decreased to 6.59 ± 3.16% after 4 days, while no activity was evident after 7 days. At 1 mg/mL, a higher inhibitory capacity was observed, with values ranging from 67.11 ± 2.64% to 54.12 ± 3.44% over the incubation time. For both the tested concentrations, the anti-glycative effect reached the highest capacity after 1 day of incubation and then progressively decreased over time, indicating a similar kinetic profile. Similarly, *A. citrodora* extract resulted in a significant reduction in Vesperlysine-like AGE formation at both 0.5 and 1 mg/mL, with a clear dose-dependent effect (Figure 2b). In contrast to the behavior observed for Argpyrimidine-like AGEs, the inhibitory effect on Vesperlysine-like AGE formation was nearly constant over the incubation time, except at the 0.5 mg/mL extract concentration when tested after 7 days, which had no activity. In fact, the extract reduced Vesperlysine-like AGEs formation from 35.25 ± 2.88% to 32.96 ± 1.46% at 0.5 mg/mL until day 4, while the inhibition linearly increased, reaching values between 62.17 ± 2.6% and 66.02 ± 1.51% at 1 mg/mL. However, its efficacy remained lower than that registered for AG.

**Figure 3:**
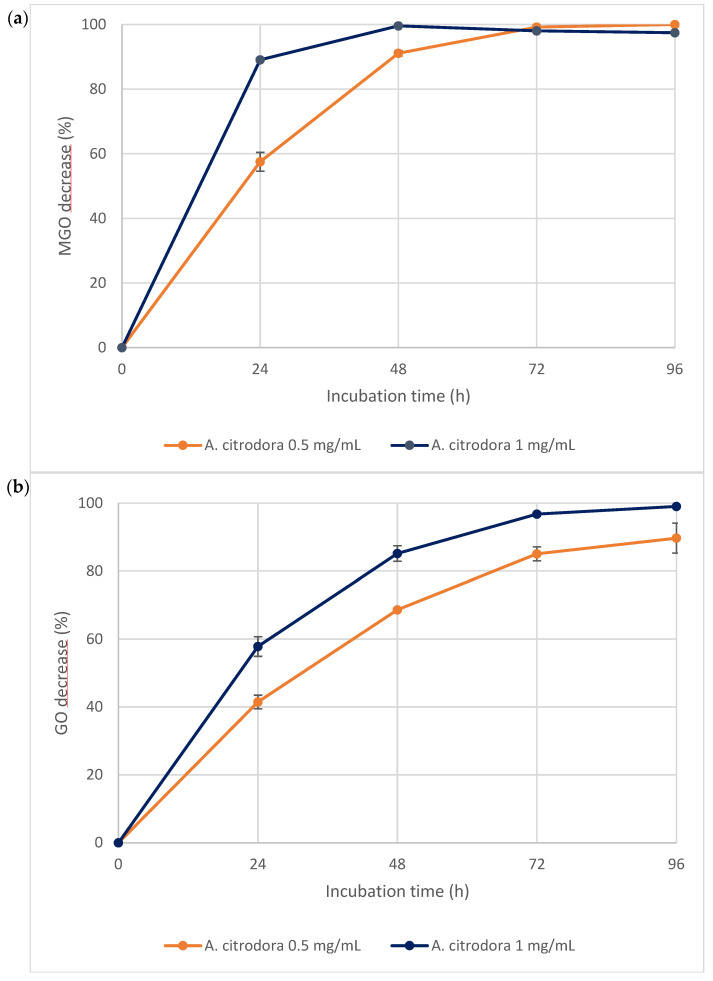
MGO (**a**) and GO (**b**) percentage trapped by *A. citrodora* extract at 0.5 mg/mL and 1 mg/mL.

To further assess the extract’s anti-glycative properties, its direct trapping effect on MGO and GO was evaluated by incubating the dicarbonyl compounds with the extract (tested at 0.5 and 1 mg/mL) for 24, 48, 72, and 96 h. A strong trapping capacity toward both MGO and GO was evident, showing a dose-dependent response and an increase in activity over time (Figure 3a,b). The extract at 0.5 mg/mL trapped nearly 100% of MGO after 72 h of incubation, while 89.67 ± 4.42% of GO was trapped after 96 h. In contrast, when tested at 1 mg/mL, MGO was completely trapped within 48 h, while GO trapping reached nearly 100% only after 96 h. However, a difference in reaction kinetics was observed between MGO and GO, since high levels of MGO trapped were already reached after 48 h, while the percentage of GO trapped increased more gradually over time. This difference could be ascribed to the nature of GO, which mainly exists in aqueous solution as a hydrated monomer that tends to polymerize easily into dimeric or trimeric structures that are in equilibrium with one another. As a result, the trapping efficiency for GO may be lower than that for MGO because less free GO was available for the reaction [49].

**Figure 4:**
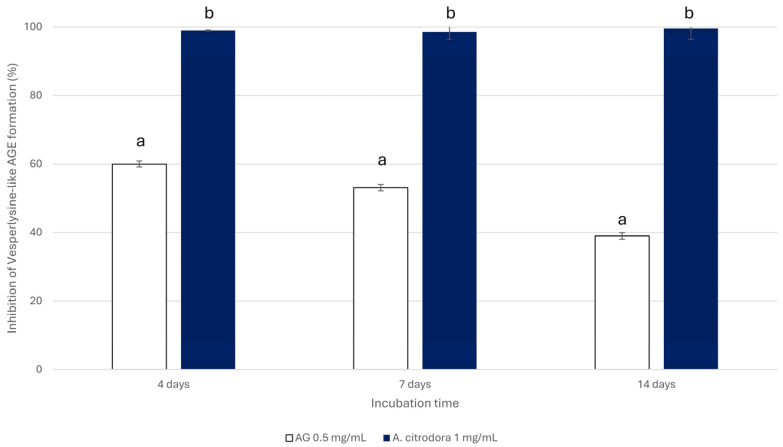
Inhibition of Vesperlysine-like AGE formation in the BSA-FRU system in the presence of *A. citrodora* extract (1 mg/mL) or AG (positive control, 0.5 mg/mL). Statistically significant differences (*p* < 0.05) between the extract and AG are represented by different superscript letters at each incubation time.

Finally, the BSA-FRU model system was used to evaluate the anti-glycative properties of the extract in the final step of the glycation process. BSA was incubated with FRU at 37 °C for 4, 7, and 14 days, according to the known kinetics of FRU-mediated glycation, to monitor the formation of Vesperlysine-like AGEs [50]. Similarly to the middle step, the extract was able to counteract the AGEs’ formation when tested at 1 mg/mL; thus, its anti-glycative properties in the final step of the glycation were evaluated only at this concentration. As reported in Figure 4, a marked reduction (higher than 97%) in Vesperlysine-like AGE content was observed at all incubation times. Notably, its activity was always higher than that of AG, whose effect progressively declined over time (60.06 ± 0.86%, 53.10 ± 0.95%, and 38.69 ± 0.95% after 4, 7, and 14 days of incubation, respectively).

In conclusion, the *A. citrodora* leaf methanolic extract exhibited high anti-glycative capacities in the middle and final steps of the glycation reaction, as well as significant trapping activity toward MGO and GO. These effects could be attributed to the composition of its secondary metabolites, which consisted mainly of iridoids glycosides, phenylethanoid glycosides, and flavonoid derivatives. In fact, secondary metabolites are well known for their ability to inhibit glycation reactions and to prevent AGE formation through different mechanisms, including via the protection of glycation sites on proteins, the reduction in oxidative stress, the trapping of reactive dicarbonyls, and the chelation of metal ions [7]. For example, verbascoside and isoverbascoside, the most abundant metabolites in the extract, and their structural components, caffeic acid and 3,4-dihydroxyphenylethanol, effectively reduced AGE formation in several in vitro models, such as BSA-GLU, BSA-galactose, and BSA-MGO. Specifically, in short-term incubations, all the compounds decreased the formation of carboxymethyllysine in the BSA-galactose system, while in the GLU model, only caffeic acid and 3,4-dihydroxyphenylethanol maintained a constant protective effect, even after a long time. The anti-glycative activity against Argpyrimidine-like AGEs was even more evident in the MGO model, where these molecules already showed high inhibitory activity at low concentrations. Based on these results, the phenolic moiety appears to play a key role in the anti-glycative properties of phenylethanoid glycoside derivatives, where the catechol group enhances their antioxidative properties thanks to its ability to act as a scavenger and to neutralize the reactive species involved in glycation processes [51]. The high activity of verbascoside was also confirmed by another study testing a methanolic extract of the aerial parts and roots of two Scutellaria species (*S. alpina* and *S. altissima*), as well as five identified secondary metabolites (luteolin, luteolin-7-glucoside, baicalin, wogonoside, and verbascoside). The extracts reduced AGE formation by approximately 50–70%, while verbascoside reduced this by 70%, displaying activity comparable to that of AG. Furthermore, a significant correlation between the extracts’ anti-glycative properties and the verbascoside content was observed [52]. Similarly, *Olea europaea* L. leaf extract, which is rich in secondary metabolites, including verbascoside, significantly reduced BSA glycation. This effect was confirmed by both fluorescence studies, which highlighted a reduction in fluorescence intensity correlated with reduced AGE formation, and gel electrophoretic analyses, indicating a reduced protein electrophoretic migration when the extract was present compared with both glycated and non-glycated BSA. This reduction was attributed to the interaction of the metabolites present in the extract with the protein, which affected its electrophoretic properties. Conversely, the increased formation of AGEs in the absence of extract led to an increase in electrophoretic mobility due to the loss of positive charges on arginine and lysine residues (the main sites involved in the glycation reaction), with a consequent variation in the BSA isoelectric point [53]. Furthermore, although no data have been reported on the anti-glycative properties of hispidulin and jaceosidin detected in *A. citrodora* extract, recent studies have highlighted the anti-glycative properties of other flavones, such as apigenin, chrysin, and luteolin, correlating their structural features with their activity. In fact, luteolin and apigenin exhibited similar AGE inhibitory effects (60.04 ± 0.52% and 59.10 ± 0.12%, respectively), whereas chrysin displayed lower activity (45.08 ± 0.10%). These findings suggested that the presence of hydroxyl groups on the B ring (as occurs in luteolin and apigenin) played a key role in modulating the ability of flavones to inhibit AGE formation [54]. Mohammadpour et al. [55] also reported that hydroxylation at C4′ and the C2–C3 double bond enhanced the anti-glycative potential of flavones, as luteolin and quercetin were more effective than naringenin (a flavanone). Moreover, docking studies revealed that flavones interacted with glycation-prone lysine and arginine residues within the “Sudlow pocket” through non-covalent interactions, preventing AGE formation [55]. Vitexin and isovitexin, isolated from *Vigna radiata*, exhibited a strong anti-glycative activity that was directly correlated with the presence of a 4′-hydroxyl group on the B ring. Similarly, iridoid glycosides have been proposed as potential anti-glycative agents acting through multiple mechanisms, including via the trapping of reactive dicarbonyl intermediates and the modulation of reactive oxygen species (ROS) formation, thereby preventing AGE formation [56]. For example, hydroxytyrosol, the main metabolite of oleuropein, trapped MGO under physiological conditions, thereby acting as a carbonyl scavenger and limiting AGE formation [57]. Loganic acid directly inhibited the in vitro BSA glycation induced by both FRU and GLU, demonstrating its potential as an anti-glycative agent [56]. Therefore, the observed anti-glycative properties for *A. citrodora* extract could likely be the result of a synergistic effect among the different metabolites present, even if the most abundant metabolites are verbascoside and isoverbascoside, which are well known in the literature for their anti-glycative effect.

### 3.3. Investigation into the Effect of In Vitro Static Digestion on A. citrodora Extract

In vitro static digestion of *A. citrodora* extract was performed to assess the impact of gastrointestinal conditions on the identified metabolites as well as on its anti-glycative properties. Therefore, in the first step, preliminary studies on bioaccessibility were conducted, and then the anti-glycative properties of the bioaccessible fraction were evaluated.

#### 3.3.1. Evaluation of the A. citrodora Extract Bioaccessibility

**Figure 5:**
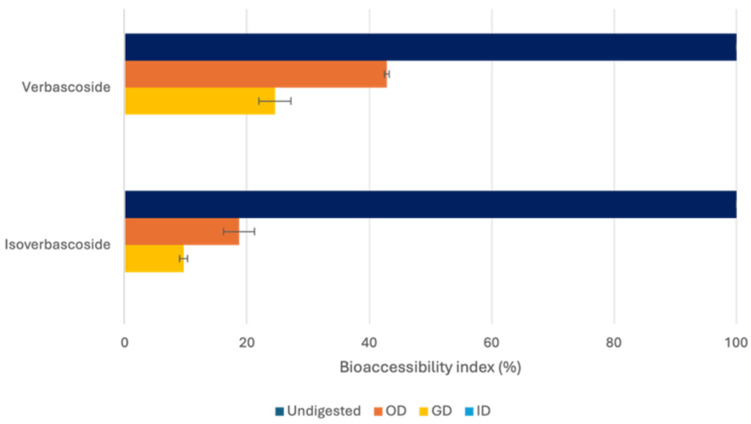
Bioaccessibility index (%) of verbascoside and isoverbascoside after oral digestion (OD), gastric digestion (GD), and intestinal digestion (ID).

The bioavailability of secondary metabolites is affected by multiple factors, including polarity, molecular mass, interactions with the food matrix, digestive processes, and absorption mechanisms. A key factor for intestinal absorption is bioaccessibility, defined as the fraction of a compound released from the food matrix and potentially available for absorption. Bioaccessibility can be affected by variables such as the solubility and stability of metabolites during digestion, which are, in turn, influenced by pH changes, enzymatic hydrolysis, and mechanical disintegration. To investigate the extract bioaccessibility, in vitro static digestion models simulating human gastrointestinal conditions represent a practical and effective approach for obtaining preliminary information about changes in the metabolic profile at different digestion phases, while dynamic digestion models represent potential more predictive advances for validating the preliminary results before performing animal experiments [58]. In this study, *A. citrodora* extract (1 mg/mL) was subjected to in vitro static digestion, and the supernatants collected from the oral, gastric, and intestinal phases were analyzed by RP-HPLC-ESI-DAD-MS^n^ to monitor changes in the metabolite profile, as reported in Table 2 and Figure 5. In parallel, control and blank samples were digested to evaluate the stability of the extract under the experimental conditions (without enzymes) and to exclude potential interferences deriving from the digestion medium, respectively.

Among the metabolites identified in the *A. citrodora* extract, only verbascoside and isoverbascoside were detected after in vitro digestion. The absence of the other metabolites after the oral phase, were likely due to progressive dilution during the digestive phases, resulting in concentrations below the LOD or because of low stability or solubility in the gastric/intestinal fluids. The verbascoside concentration (7.40 ± 0.84 µg/mL in the undigested extract) progressively decreased during the oral (3.16 ± 0.03 µg/mL) and gastric (1.38 ± 0.19 µg/mL) phases, becoming undetectable after the intestinal phase. In the oral control sample, a decrease in verbascoside content (3.57 ± 0.08 µg/mL) was also registered and was mainly related to the dilution factor (1:1, *v*/*v*) occurring in the oral phase; in contrast, in the digested sample, a higher reduction was observed, probably due to an interaction with α-amylase, which could be inhibited by verbascoside, as has been reported in the literature [59]. In contrast, no significant differences were observed between the gastric control and digested samples, where the reduction appeared to be largely correlated with the dilution, suggesting verbascoside stability under gastric conditions and in the presence of digestive enzymes. However, this compound proved highly unstable under intestinal conditions, becoming undetectable. Overall, verbascoside exhibited good bioaccessibility values at the end of the oral step (42.84 ± 0.4%), which then decreased (24.56 ± 2.6% and 0% after the gastric and intestinal phases, respectively).

Similarly, isoverbascoside (4.72 ± 0.29 VE µg/mL in the undigested extract) concentration decreased to 1.16 ± 0.12 VE µg/mL and 0.45 ± 0.036 VE µg/mL after the oral and gastric phases, respectively, and the metabolite was not detected at intestinal level. This reduction was higher than that expected based on the dilution factor. In this case, no significant differences were observed between the control and digested samples in either the oral or gastric phase, suggesting that isoverbascoside was not susceptible to enzymatic activity but was unstable under these experimental conditions. Its bioaccessibility values were estimated to be 18.71 ± 2.5%, 9.70 ± 0.66%, and 0% after oral, gastric, and intestinal digestion phases, indicating an overall lower bioaccessibility compared with verbascoside.

These findings were consistent with those previously reported by Cardinali et al. [60], who observed higher bioaccessibility for verbascoside than isoverbascoside. However, both compounds were markedly unstable during the intestinal digestion, suggesting degradation under the alkaline pH conditions or hydrolysis, which could likely lead to the release of a degradation product such as caffeic acid (not detected in this study) [61]. Similarly, Zhou et al. [62] reported that the content of verbascoside in *Osmanthus fragrans* Lour. flowers decreased with increasing pH, resulting in low bioaccessibility after intestinal digestion. A comparable trend was also observed for verbascoside in olive leaf extract subjected to in vitro digestion, further confirming its instability under intestinal conditions [63]. In fact, verbascoside could undergo different degradation pathways at alkaline pH, including a hydrolysis reaction yielding caffeic acid and hydroxytyrosol, isomerization processes, or oxidative decomposition due to auto-oxidation [64]. These chemical transformations may explain the undetectable levels after intestinal digestion and highlight the key role of pH in compound instability.

#### 3.3.2. Anti-Glycative Properties of the A. citrodora Extract Bioaccessible Fraction

The anti-glycative activity of the bioaccessible fraction (digested intestinal supernatant sample) was evaluated at 0.125 mg/mL, representing the extract concentration in the intestinal phase, to study the impact of the digestion process on the bioactivity of the *A. citrodora* extract. Since the undigested extract had previously shown high dicarbonyl compound trapping capacity and high anti-glycative activity in the final step of glycation, the ability of the bioaccessible fraction to trap MGO and GO and to inhibit the formation of Vesperlysine-like AGEs in the BSA-FRU system was investigated. Furthermore, intestinal control and blank samples were tested to evaluate the influence of the experimental conditions and digestion medium on the bioactivity of the digested extract.

**Figure 6:**
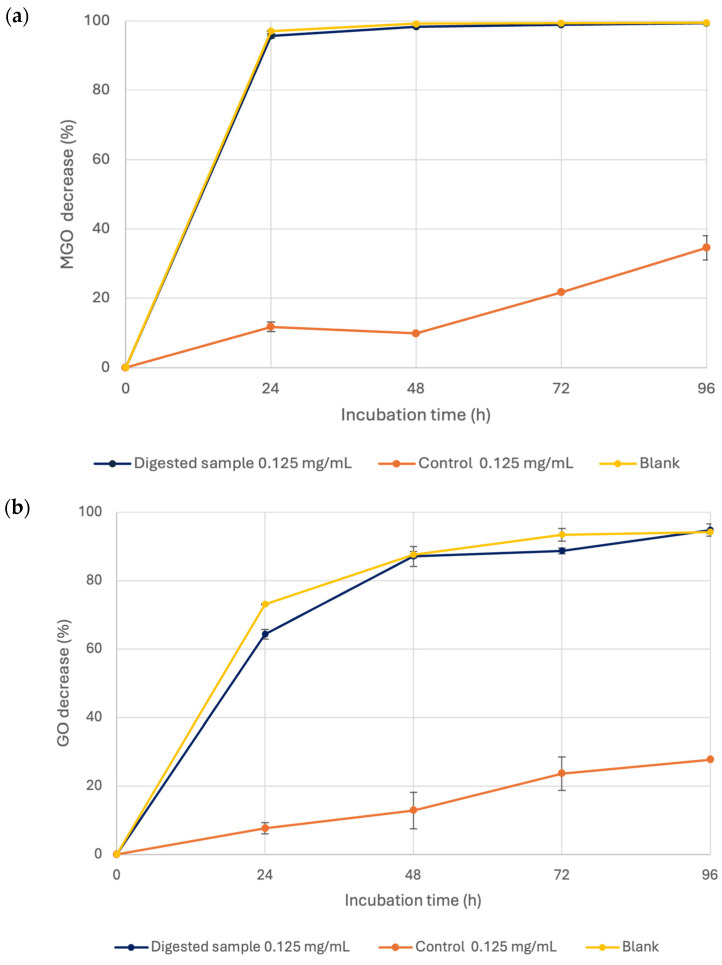
MGO (**a**) and GO (**b**) trapping capacity of digested, control (0.125 mg/mL), and blank samples after the intestinal digestion phase.

As shown in Figure 6a,b, the digested intestinal sample already had a high MGO trapping capacity after 24 h (95.66 ± 0.59%), reaching almost 100% after 96 h. In contrast, for GO, a progressive increase in trapping capacity was observed with incubation time, from 64.30 ± 1.39% to 94.75 ± 1.79%. A similar trend was observed for the blank, which was not significantly different from the digested sample. These results suggested that the high trapping activity observed in the digested sample may be largely attributed to the presence of digestive enzymes, which could interact with dicarbonyl compounds and generate potential false positives, as previously reported by Ferron et al. [15]. However, the control maintained a partial trapping capacity toward both MGO and GO, with an increase in activity over time (from 11.78 ± 1.40% to 34.58 ± 3.49% for MGO and from 7.66 ± 1.66% to 27.71 ± 0.05% for GO). These data indicated that the digested sample still partially preserved the trapping activity, even if only at a low level, which is likely due to the formation of undetected bioactive metabolites generated under alkaline conditions, whose detection could require more sensitive analytical techniques, such as LC-HRMS.

**Figure 7:**
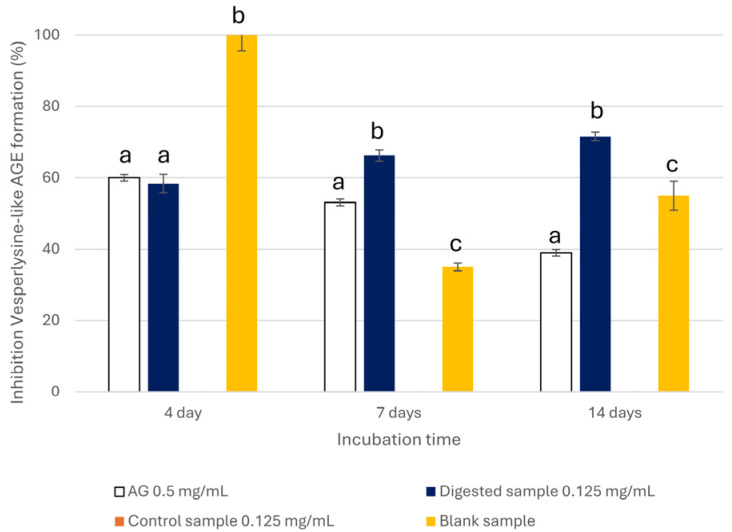
Inhibition of Vesperlysine-like AGE formation in the BSA-FRU system registered for digested, control (0.125 mg/mL), and blank samples after the intestinal phase. AG was used as a positive control (0.5 mg/mL). Statistically significant differences (*p* < 0.05) between the sample and AG were represented by different superscript letters at each incubation time.

As regards the extract’s ability to inhibit AGE formation in the BSA-FRU system (Figure 7) after digestion, this was reduced to 58.37 ± 2.56% after 1 day of incubation. Conversely, the blank completely inhibited the glycation reaction. After 4 and 7 days, the inhibition increased to 66.22 ± 1.55% and 71.58 ± 1.16%, respectively, with values higher than those registered for the blank (35.32 ± 1.08% and 55.01 ± 4.09%). These data suggested that the activity observed at the beginning of the monitoring period (1 day) was mainly due to the presence of digestive enzymes, while for longer periods, the higher activity observed in the digested sample compared to the blank one indicated that the digested sample still partially preserved the inhibitory effect on AGE formation. The absence of activity in the control also indicates that the combination of enzymes and undetected metabolites may enhance the anti-glycative properties of the digested extract.

### 3.4. Molecular Docking and MM-GBSA Analysis

**Figure 8:**
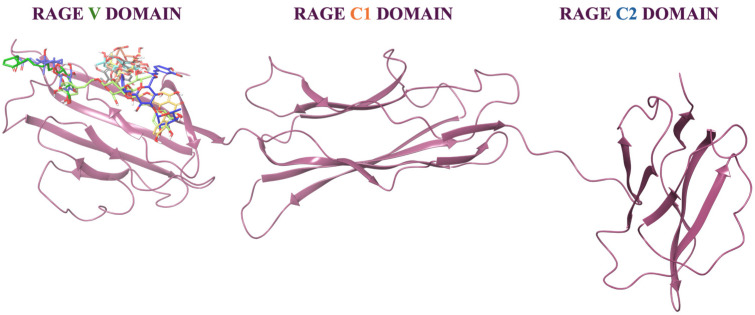
Overall structure of the hRAGE C1, C2, and V domains (purple cartoon). 3D representation of the identified metabolites in complex with the RAGE V domain. The ligands are shown as different colored carbon sticks.

Molecular docking analysis (Figure 8) revealed that all the identified metabolites were able to recognize and bind to the V domain of the RAGE. In detail, the metabolites established different interactions with the V domain, including hydrogen bonds and hydrophobic contacts, which may contribute to the stability and affinity of the ligand–receptor complex.

Moreover, to investigate the thermodynamic profile and stability of the *A. citrodora* secondary metabolites in complex with RAGE, Molecular Mechanics Generalized Born Surface Area (MM-GBSA) calculations were performed. For each compound present in the extract, the top-ranked docking pose was subjected to a post-docking analysis through energy minimization and, subsequently, to binding free-energy calculations (MM-GBSA). The results are reported in Table 3.

**Figure 9:**
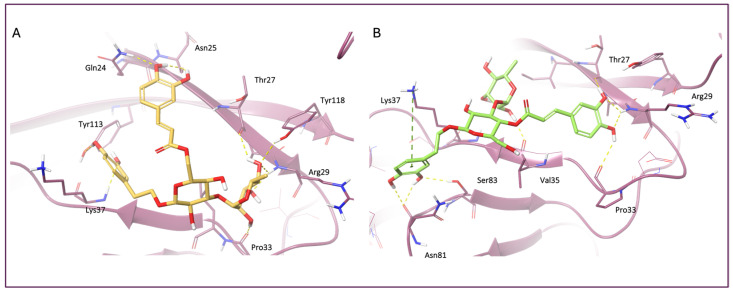
3D representation of (**A**) isoverbascoside (yellow carbon sticks) and (**B**) verbascoside (green carbon sticks) in complex with the RAGE V domain. The RAGE V domain is represented as a purple cartoon, and the amino acids engaged in pivotal contacts with the ligand are represented as purple carbon sticks. Salt bridges, pi-cation and hydrogen bond interactions are represented by magenta, green, and yellow dashed lines, respectively.

Overall, the calculated binding free energies confirmed that all the analyzed metabolites were able to form stable complexes with the RAGE V domain. Among the investigated compounds, isoverbascoside (Figure 9A) had the most favorable ΔG binding value (−85.38 kcal/mol), indicating a strong and energetically favorable interaction with the receptor. Verbascoside (Figure 9B) also displayed notably low binding free-energy values, suggesting good binding stability.

## 4. Conclusions

In this study, the anti-glycative properties of *A. citrodora* leaf extract were evaluated at different steps of the glycation reaction, along with a preliminary investigation of its bioaccessibility through simulated in vitro static digestion. The extract showed high anti-glycative capability, as it was able to significantly inhibit AGE formation at the middle and end steps of the glycation reaction. Furthermore, a significant reduction in free MGO and GO was observed, indicating a very good trapping capacity. These properties could be attributed to the presence of several bioactive secondary metabolites, including flavones, iridoid glycoside, and phenylethanoid glycosides. Molecular modeling analyses revealed the ability of these metabolites to interact with the V domain of RAGE, which is also supported by their good theoretical binding stability. However, simulated in vitro digestion highlighted a general decrease in the metabolites’ concentration, mainly in the alkaline conditions of the intestinal phase, as observed for verbascoside and isoverbascoside, resulting in poor bioaccessibility. However, the bioaccessible fraction still maintained partial anti-glycative activity, likely due to the presence of undetected metabolites.

These preliminary data suggested that *A. citrodora* extract could be considered a promising source of bioactive metabolites with in vitro anti-glycative properties, as reported here for the first time. However, important limitations remain that require further investigation, including the extension of in vitro static digestion studies to a dynamic model, to validate the results under conditions more predictive of a similar-physiological environment. In addition, the development of a suitable carrier system is needed to encapsulate the extract and improve metabolites’ stability under gastrointestinal conditions, thereby increasing their bioaccessibility and preserving their biological activity. Further studies could be also required to better characterize the undetected metabolites formed during digestion, but this limitation could be partially solved by an optimized formulation that makes higher metabolite concentrations bioaccessible. Finally, in vivo studies will be necessary to confirm the observed anti-glycative properties. All these future directions will be useful to overcome the current limitations and to further support the potential use of *A. citrodora* extract as a food ingredient in dietary supplements, responding to the growing need to develop new natural anti-glycative agents for the management of AGE-related diseases.

## Figures and Tables

**Table 1:** Retention time (Rt), UV-Vis, MS, and MS^n^ data of compounds identified in *A. citrodora* extract.

Compound n.#	Rt (Min)	UV-Vis λ_max_	[M-H]^−^ (*m*/*z*)	Fragmentation MS^n^ (% Base Peak)	Identification	References
1	7.85	238	437 ^1^	MS^2^[436]: 391 (100) MS^3^[391]: 391 (46), 229 (24), 211 (8), 185 (30), 167 (100), 149 (45)	Shanzhiside	[32,33]
2	8.34	238	419 ^1^	MS^2^[418]: 373(100) MS^3^[373]: 211 (21), 167 (18), 149 (68), 123 (100)	Geniposidic acid	[34,35]
3	8.87	238	421 ^1^	MS^2^[420]: 375 (100) MS^3^[375]: 213 (54), 169 (67), 151 (100), 125 (57)	Loganic acid	[36]
4	12.53	240	433 ^1^	MS^2^[433]: 387 (100), 225 (13)	Rehmaionoside C	[37,38,39]
5	12.91	240	387	MS^2^[387]: 207 (50), 163 (100)	Tuberonic acid glucoside	[40,41]
6	16.07	220, 250, 295, 330	623	MS^2^[623]: 461 (100)	Verbascoside ^2^	[42]
7	16.53	220, 250, 295, 330	623	MS^2^[623]: 461 (100)	Isoverbascoside	[42]
8	17.96	215, 275, 335	299	MS^2^[299]: 284 (100)	Rhamnocitrin	[43]
9	18.15	227, 335	329	MS^2^[329]: 314 (100), 299 (2)	Jaceosidin	[44,45]

^1^ formic acid adduct; ^2^ compared with standard compound.

**Table 2:** Quantification of bioaccessible verbascoside and isoverbascoside in each digestion phase.

Compound	Undigested Sample (µg/mL)	OC (µg/mL)	OD (µg/mL)	GC (µg/mL)	GD (µg/mL)	IC (µg/mL)	ID (µg/mL)
Verbascoside	7.40 ± 0.84 ^a^	3.57 ± 0.08 ^b^	3.16 ± 0.03 ^c^	1.81 ± 0.15 ^d^	1.38 ± 0.19 ^d^	nd	nd
Isoverbascoside	4.72 ± 0.29 ^a^	1.20 ± 0.04 ^b^	1.16 ± 0.12 ^b^	0.55 ± 0.036 ^c^	0.45 ± 0.03 ^c^	nd	nd

OC = oral control sample; OD = oral digested sample; GC = gastric control sample; GD = gastric digested sample; IC = intestinal control sample; ID = intestinal digested sample; nd = not detected; different letters on the same line indicate significant differences (*p* < 0.05). Isoverbascoside was quantified as VE µg/mL.

**Table 3:** Docking score and MMGBSA values calculated for the studied metabolites vs. RAGE V domain. The values were reported in kcal/mol.

Compounds	Docking Score	MM-GBSA ΔG Bind
Isoverbascoside	−5.98	−85.38
Verbascoside	−5.37	−67.68
Loganic acid	−5.14	−63.68
Tuberonic acid glucoside	−6.09	−63.51
Rehmaionoside C	−4.28	−45.93
Geniposidic acid	−5.47	−45.06
Shanzhiside	−5.71	−44.82
Rhamnocitrin	−4.57	−44.12
Jaceosidin	−4.75	−34.88

## Data Availability

The original contributions presented in this study are included in the article. Further inquiries can be directed to the corresponding author.
